# Hip Joint Segmentation From Magnetic Resonance Imaging to Generate 3-Dimensional Models to Assess Femoroacetabular Impingement Syndrome

**DOI:** 10.1016/j.eats.2025.103714

**Published:** 2025-07-16

**Authors:** Jesus E. Cervantes, Steven P. Mell, Shane J. Nho, Catherine Yuh

**Affiliations:** Section of Young Adult Hip Surgery, Division of Sports Medicine, Department of Orthopedic Surgery, Rush Medical College of Rush University, Rush University Medical Center, Chicago, Illinois, U.S.A.

## Abstract

Accurate assessment of anatomic structures is essential for surgical planning. This is especially relevant for hip arthroscopy for femoroacetabular impingement syndrome. Beyond standard radiographs, 3-dimensional (3D) imaging often is required for preoperative assessment. Although computed tomography provides quick results with exceptional visualization of bone, magnetic resonance imaging (MRI) eliminates the exposure of ionizing radiation and provides superior soft-tissue visualization. This study demonstrates a reproducible process to generate high-quality 3D hip models from MRI using 3D Slicer to advance femoroacetabular impingement syndrome research and clinical decision-making. A 3-T MRI of the hip without contrast was imported into 3D Slicer, specifically focusing on T1 Vibe sequences. The Digital Imaging and Communications in Medicine file was deidentified. Segmentation began by isolating the femur and pelvis along the axial, coronal, and sagittal planes. A preliminary “coarse” segmentation was generated using the "Grow from Seeds" function, which is an automated region growth based on manual inputs. For precise segmentation, boundaries of the segmentation were refined. Once segmentation was complete, an initial 3D model was generated, which was then smoothed.

Accurate anatomic assessment is vital in surgical planning and decision making, particularly in complex pathomorphologic cases of femoroacetabular impingement syndrome (FAIS). Hip arthroscopy is a preferred surgical intervention for FAIS. The assessment of FAIS typically begins with standard 2-dimensional (2D) radiographs, allowing for the measurement of the lateral center-edge angle and alpha angle.^1^ However, 2D radiographs have inherent limitations, including inability to completely capture spatial relationships of the complex morphologic variations of FAIS.^2^ Reliance on 2D radiographics may lead to inaccurate assessment and planning.^3^

To address these challenges, 3-dimensional (3D) imaging often is required for comprehensive assessment and preparation for surgical intervention.^4^, ^5^, ^6^ Among available 3D imaging techniques, computed tomography offers rapid and exceptional visualization of bone structure anatomy.^7^ However, its use involves the exposure of ionizing radiation, raising concerns if frequently used, particularly in younger populations.^8^ In contrast, magnetic resonance imaging (MRI) offers significant advantages by eliminating exposure of ionizing radiation while providing superior visualization of soft-tissue pathology, such as labral tears and cartilage damage.^9^ However, lower spatial resolution and bone visualization are limitations of MRI.

The accessibility of advanced imaging software, such as 3D Slicer, continue to improve MRI utility in orthopaedic surgery and research.^10^ This study uses 3D Slicer software to reconstruct precise 3D models of the hip joint through segmentation and smoothing processes for research and clinical purposes (Video 1). The purpose of this Technical Note is to describe a reproducible process to reconstruct high-quality 3D models of the hip joint from MRI sequences in 3D Slicer software, with the intent to advance FAIS research towards improving clinical decision-making.

## Surgical Technique

### Importing DICOM Files and Deidentification

**Fig 1:**
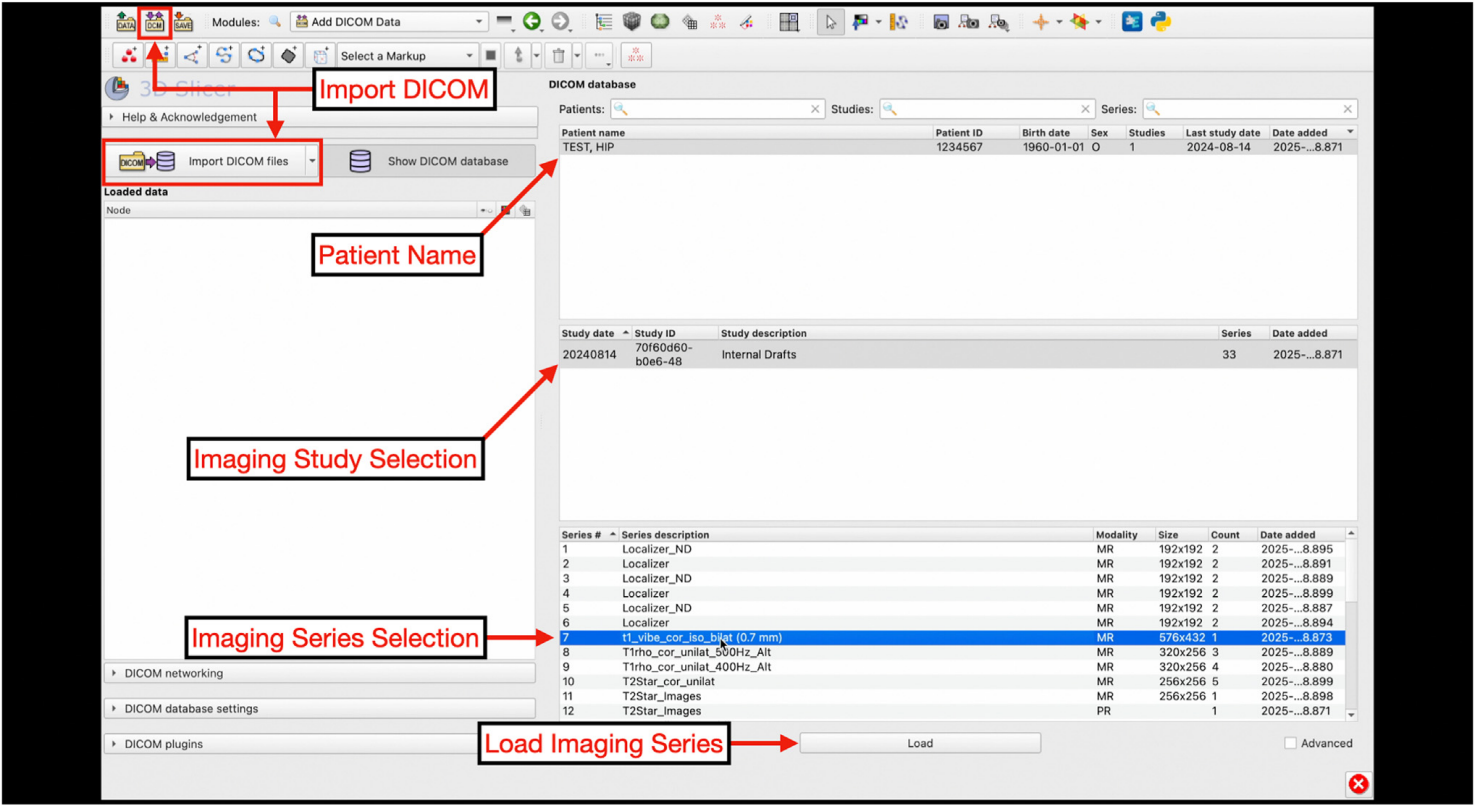
Interface of the “Add DICOM Data” module on 3D Slicer software. This is where magnetic resonance imaging DICOM files can be uploaded by clicking the “Import DICOM files” button for 3-dimensional reconstruction. The module allows users to select the patient name, imaging study, and imaging series to load. (DICOM, Digital Imaging and Communications in Medicine.)

**Fig 2:**
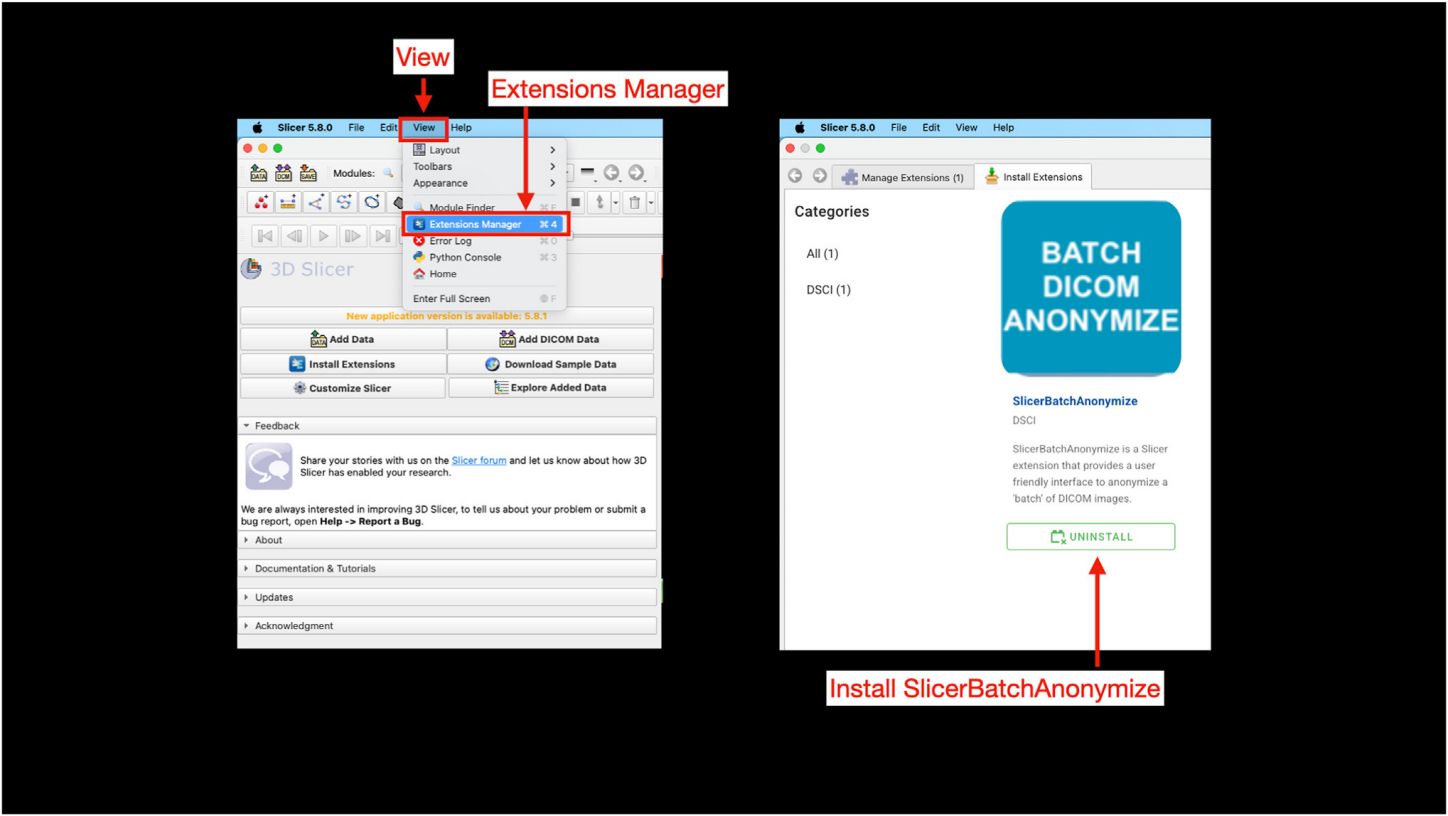
Installation of the “SlicerBatchAnonymize” extension. The “SlicerBatchAnonymize” extension can be downloaded by selecting view in the toolbar and selecting extensions manager. This will open a new tab where the user can search for and install “SlicerBatchAnonymize.”

**Fig 3:**
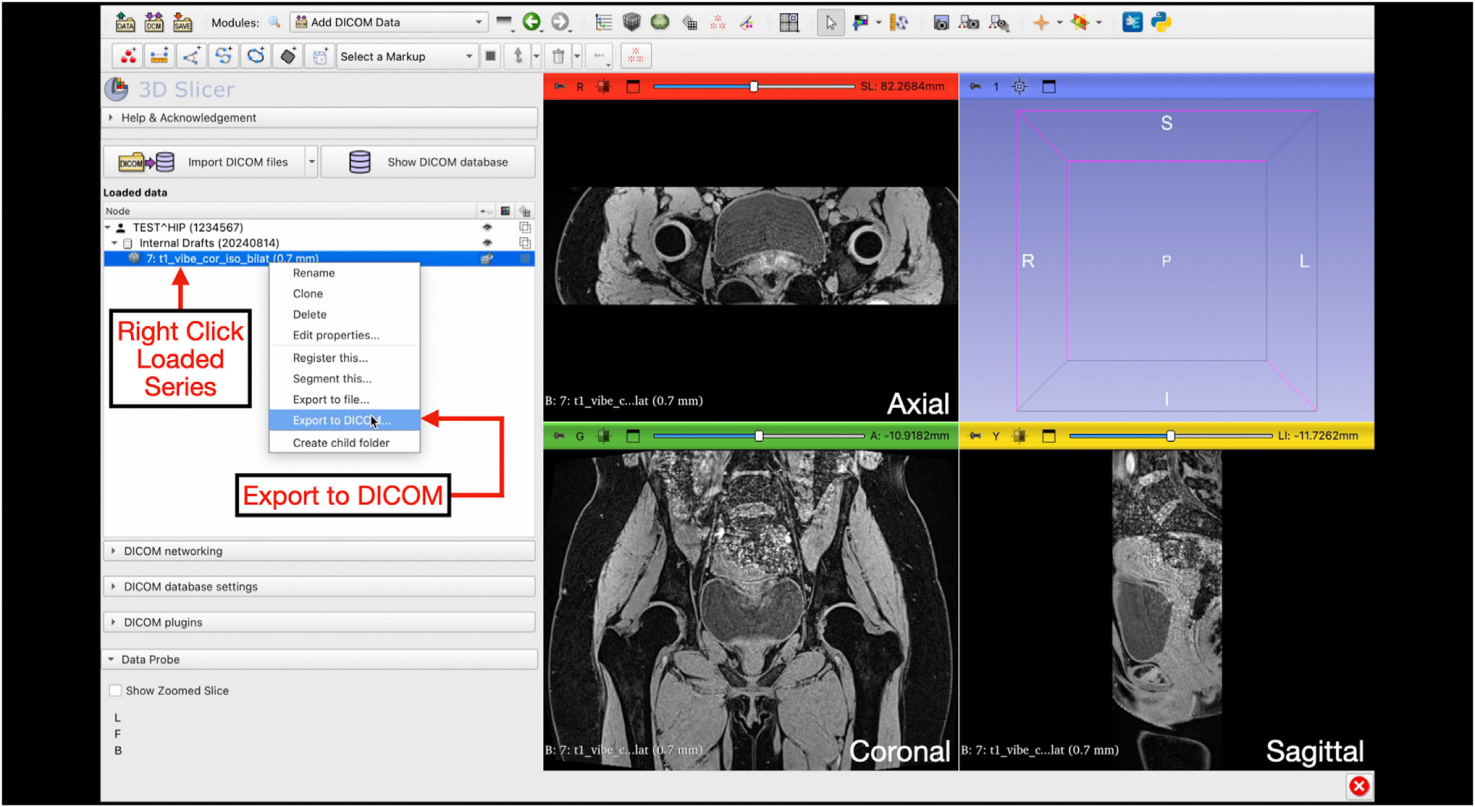
Exporting loaded DICOM in the “Add DICOM Data” module on 3D Slicer. By right clicking the loaded imaging series and exporting it as a DICOM, users can use this before deidentification. The axial, coronal, and sagittal planes of the loaded DICOM will appear in the 4-panel view. (DICOM, Digital Imaging and Communications in Medicine.)

**Fig 4:**
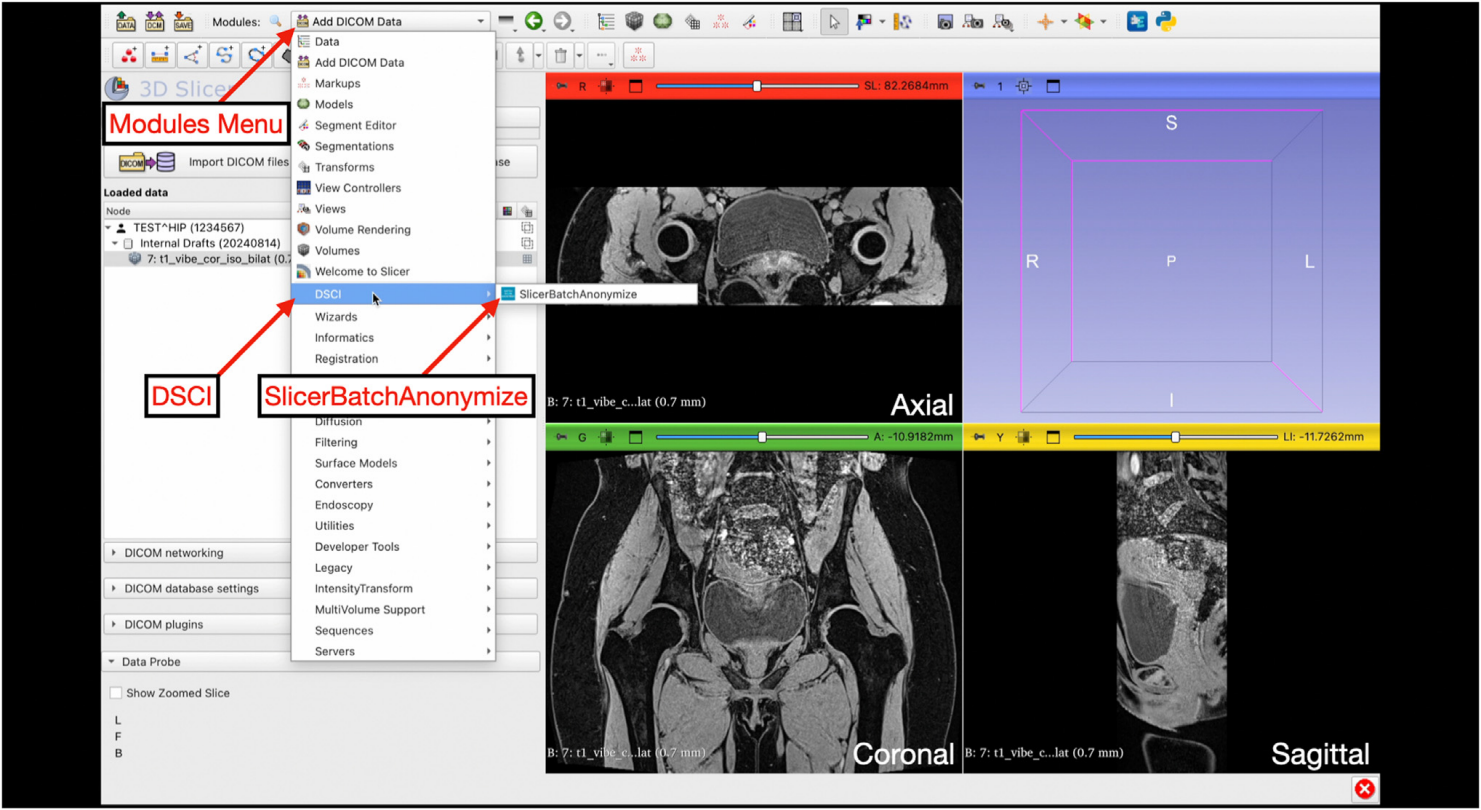
Accessing “SlicerBatchAnonymize” under the “DSCI” module on 3D Slicer. After exporting the loaded DICOM, deidentification starts by changing the module to “DSCI” and selecting “SlicerBatchAnonymize.” (DICOM, Digital Imaging and Communications in Medicine.)

**Fig 5:**
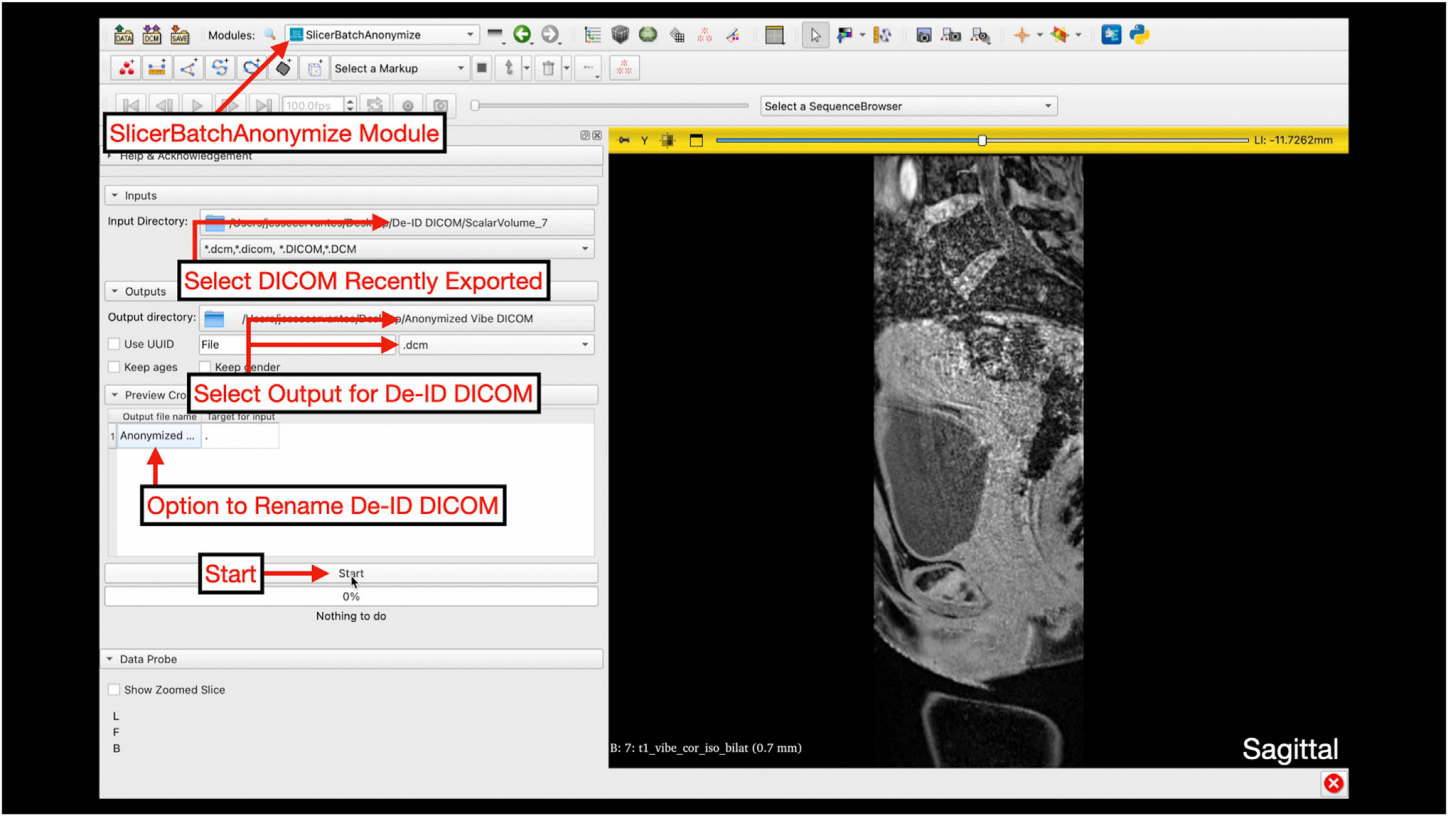
Deidentification of DICOM Series using “SlicerBatchAnonymize.” Under the “SlicerBatchAnonymize” module, the recently exported DICOM series is selected as the input. The de-identified DICOM is selected as an output using the output directory. The user must ensure the output file is selected as a .dcm. The output file name can be renamed depending on users naming conventions. The process is completed when 100% is achieved in the bar after selecting “Start.” (DICOM, Digital Imaging and Communications in Medicine.)

Digital Imaging and Communications in Medicine (DICOM) files are saved on a secure and encrypted memory drive for import to 3D Slicer (version 5.8.0; www.slicer.org). For 3-T MRI scans (3 Tesla Siemens Prisma MRI scanner; Siemens Healthineers), the postprocessing T1 Vibe sequence is used for segmentation processes (Fig 1). After importing the DICOM, the T1 Vibe sequence is exported as a DICOM and deidentified using the Data Storage, Computation, and Integration (DSCI) “SlicerBatchAnonymize” extension (version 479ac03) (Fig 2, Fig 3, Fig 4, Fig 5).

### Segmentation

**Fig 6:**
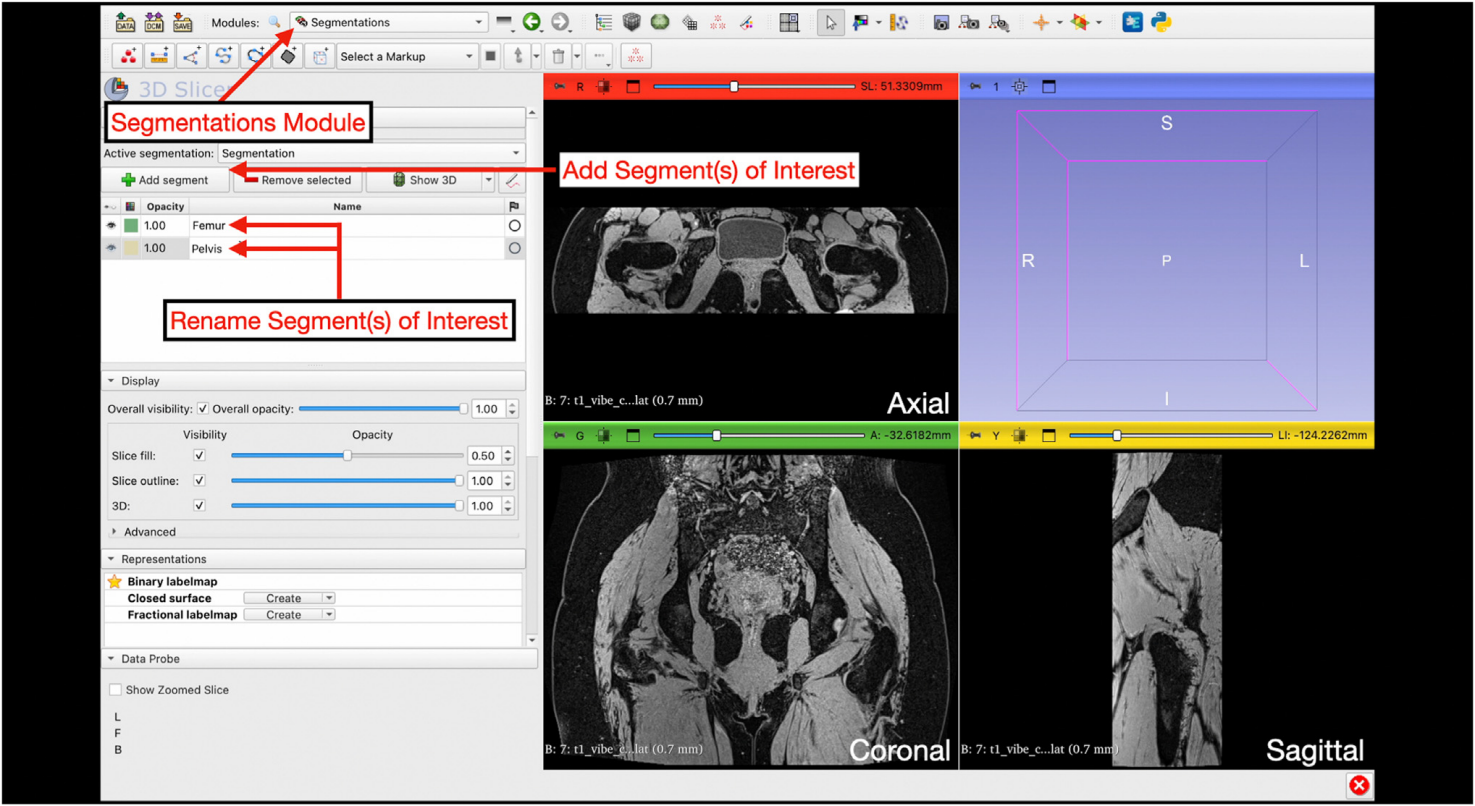
Creating segmentations. By selecting the “Segmentations” module, active segmentations can be created by selecting “Add segment.” The segment(s) of interest can then be named accordingly (e.g., “Femur,” “Pelvis”).

Segmentation is performed on the deidentified T1 Vibe sequence. Under the “Segmentations” module, new segmentations are created. For the hip joint, we used 3 separate active segmentations as masks: (1) “Femur,” (2) “Pelvis,” and (3) “NOT Bone” (Fig 6).

**Fig 7:**
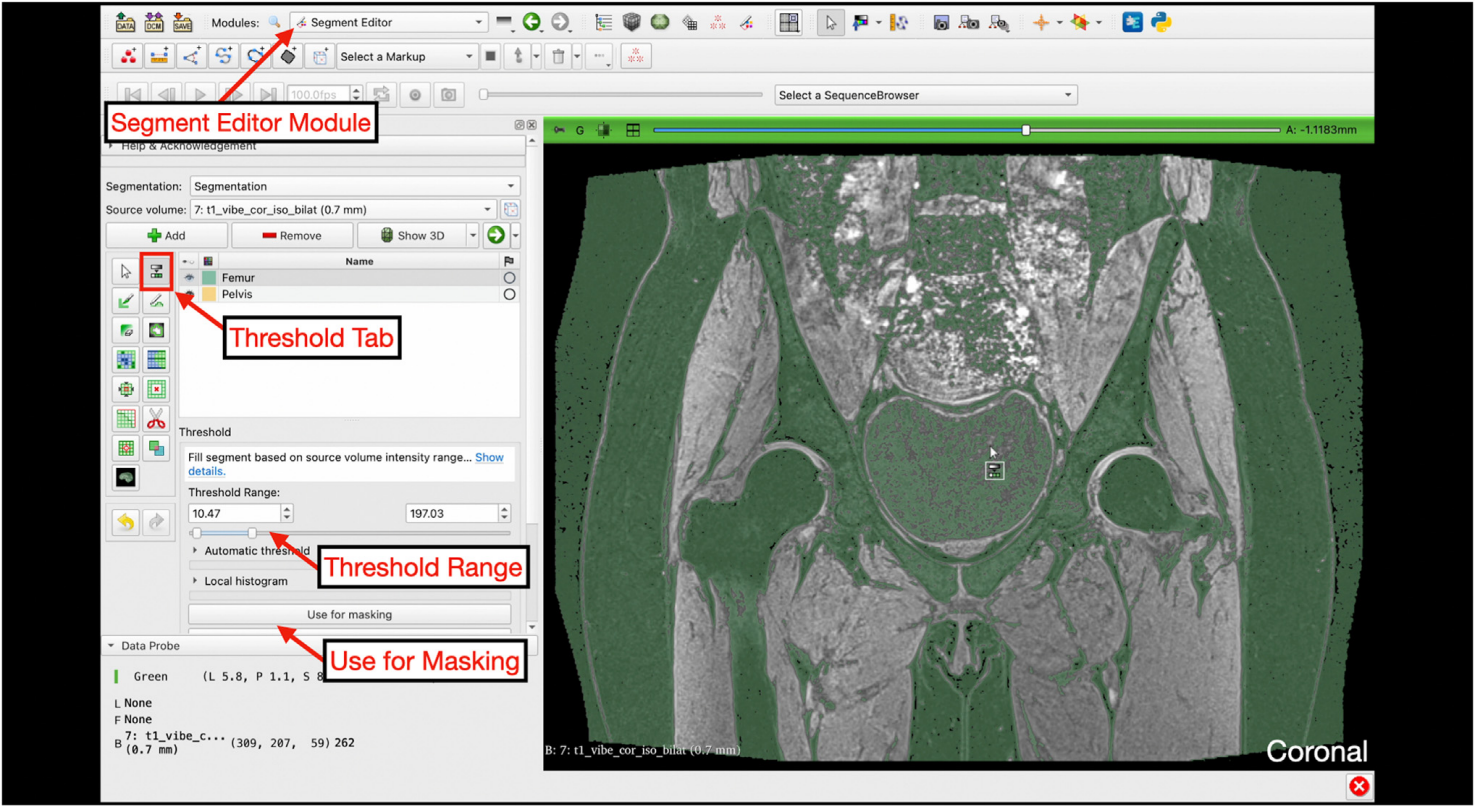
Thresholding. Under the “Segment Editor” module, the threshold tab can be selected. The user can then change the threshold range such that the entirety of bone is highlighted in green. A coronal slice of the pelvis is shown to appreciate the thresholding range isolating bone structures from nearby soft tissue. Once the appropriate range is selected, the “Use for masking” button is selected.

Under the “Segment Editor” module, the segmentation containing the newly created “Femur,” “Pelvis,” and “NOT Bone” masks is selected along with the deidentified T1 Vibe sequence as the source volume. Under the threshold option, the threshold range is set such that bone is highlighted in green but soft tissue is not. This range is used for masking and is adjustable during painting (Fig 7).

**Fig 8:**
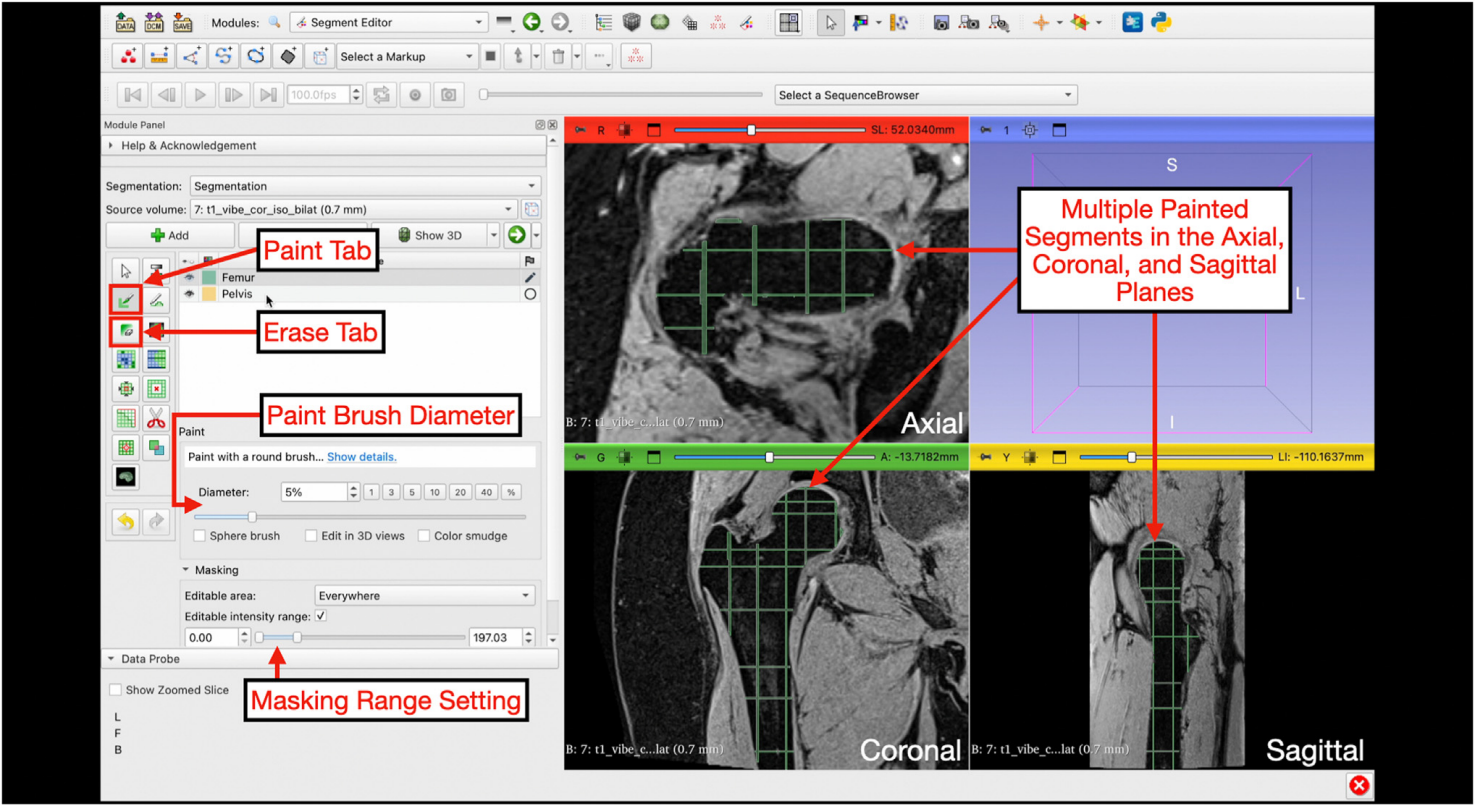
Painting and erasing the segmentations. Under the “Segment Editor” module, the paint tab can be selected. This is where the user will begin segmenting on the actual magnetic resonance imaging slices. The diameter of the paint brush can be adjusted as well as the thresholding range under the paint tab. The segment of interest is selected and painted along axial, coronal, and sagittal planes multiple times. The 4-panel view is shown with multiple slices of the proximal femur segmented in green along the axial, coronal, and sagittal planes. The erase tab has identical controls to erase painted areas.

**Fig 9:**
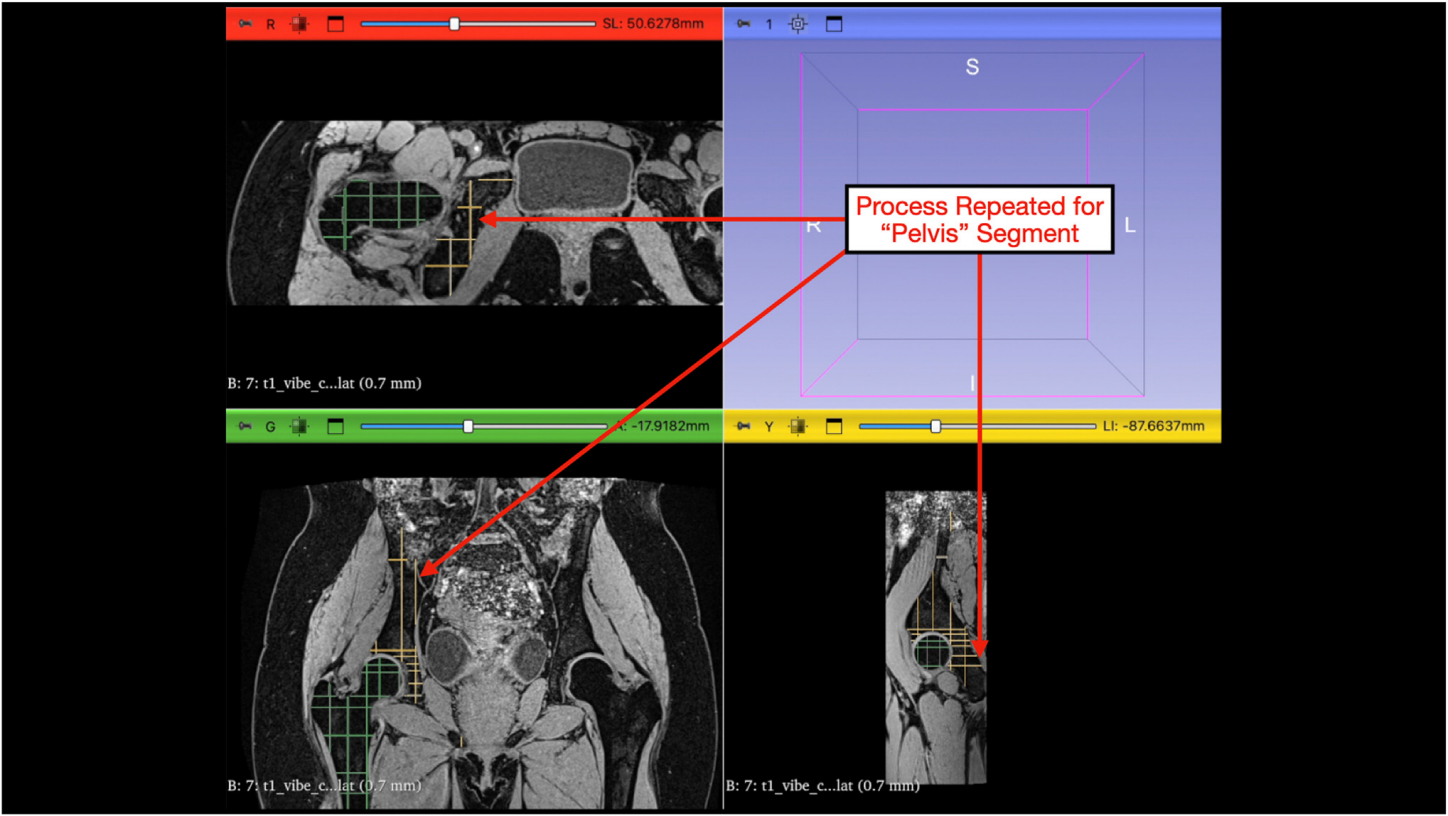
Repeating the process for the pelvis segmentation. The segment of interest, in this case the pelvis, is selected and painted along axial, coronal, and sagittal planes multiple times. The 4-panel view is shown with multiple slices of the hemipelvis segmented in yellow along the axial, coronal, and sagittal planes.

**Fig 10:**
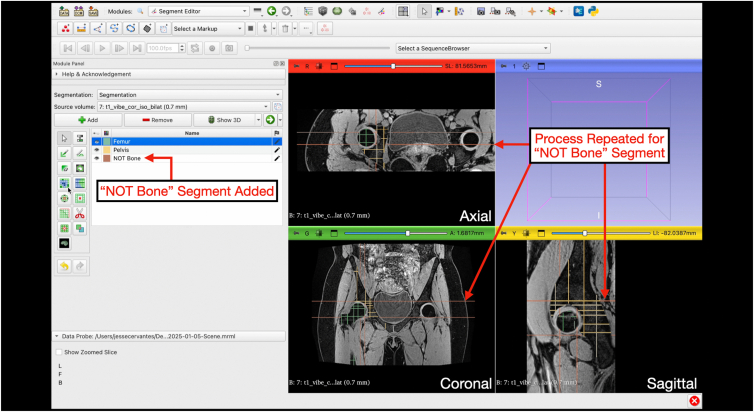
Repeating the process for the “NOT Bone” segmentation. The segment of interest, in this case the “NOT Bone,” is added as a new segment and painted along axial, coronal, and sagittal planes multiple times. The 4-panel view is shown with multiple slices of the “NOT Bone” segmentation in brown along the axial, coronal, and sagittal planes.

Using a combination of the paint and eraser tools, the “Femur” and “Pelvis” masks can be painted within the boundaries of the bone cortex defined by the masks. The diameter of the paint and eraser tools can be changed such that pixels outside of the bone are not highlighted (Figs 8 and 9). Once completed, the “NOT Bone” mask is then selected and the paint tool is used to highlight the surrounding tissue (Fig 10). This process allows for only tissue with similar contrast to bone to be highlighted under the “Femur” and “Pelvis” mask while excluding other tissue under the “NOT Bone” segmentation.

This is repeated in all planes on at least 3 slices, focusing on anatomic areas of difficulty, including the intertrochanteric crest and femoroacetabular joint space. It is recommended to save all work before starting a new slice. When saving files initially, all files should be selected and saved into a designated directory. During subsequent saves, only files modified should be selected, to ensure duplicate files are not generated. 3D Slicer includes a button at the top of the “Save Scene and Unsaved Data” dialog box that selects only the modified files.

### Grow From Seeds

**Fig 11:**
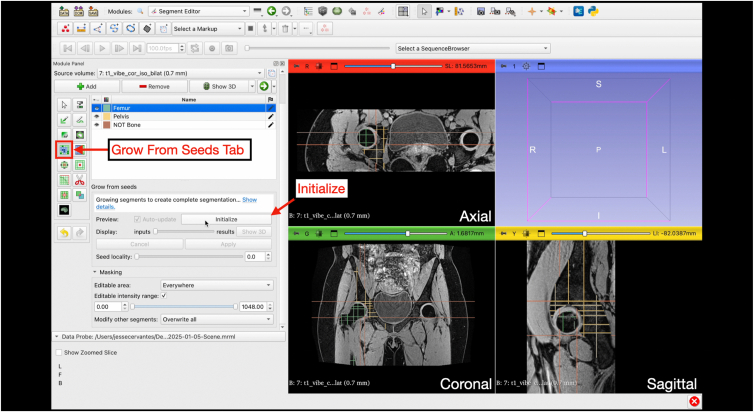
Grow from seeds. Once the user has completed the initial segmentation painting, the grow from seeds tab is selected under the “Segment Editor” module. By selecting “Initialize,” the software will use the painted segments to complete the segmentation. This step produces a course model prior to cleaning and smoothing steps.

**Fig 12:**
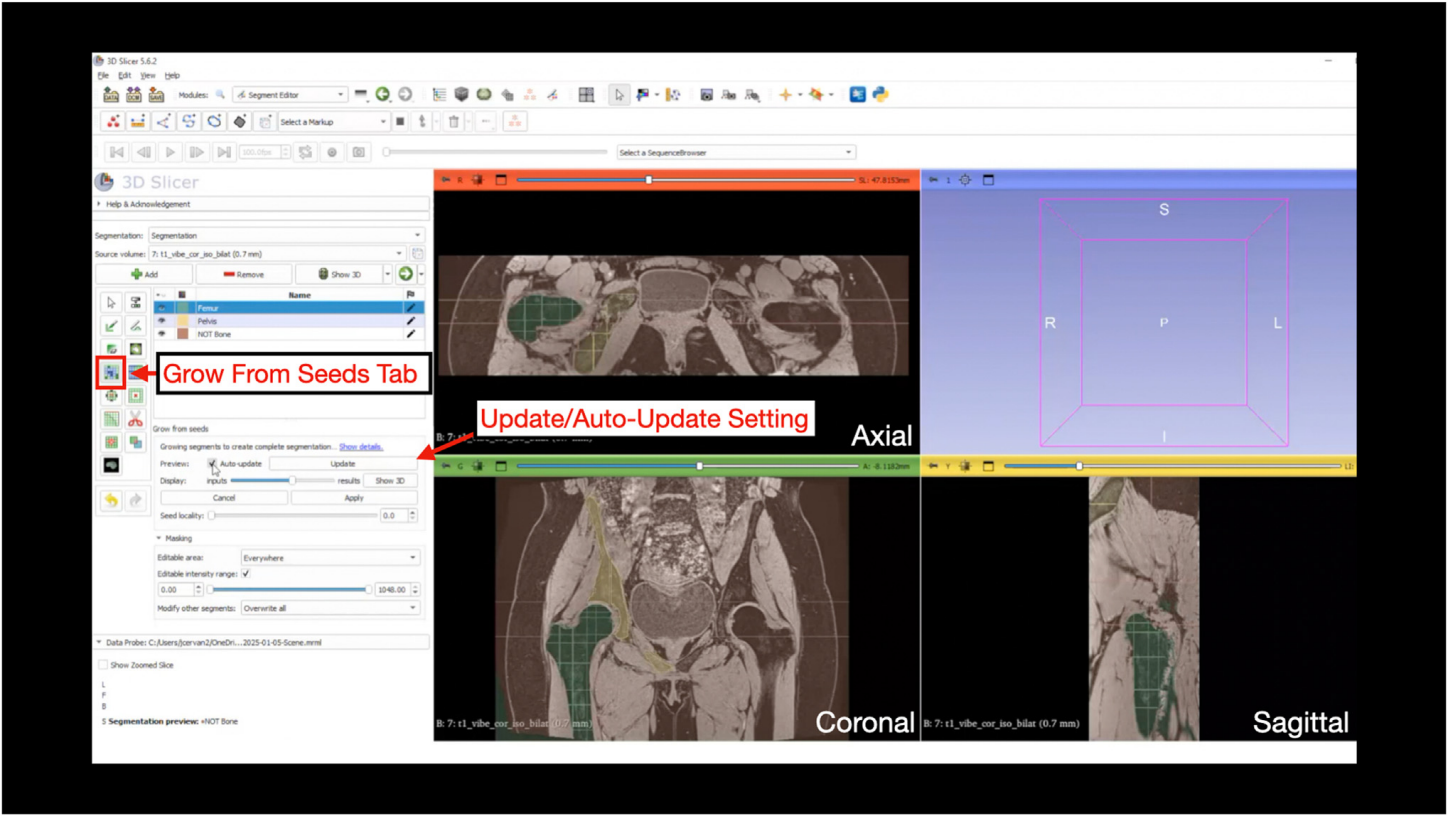
Painting and erasing the course 3-dimensional model. In the grow from seeds tab, the “auto-update” box can be selected. This allows the user to make painting and erasing edits to the course model while allowing the software to update the segmentations. The 4-panel view is shown highlighting the course segmentation generated following grow from seeds in the axial, coronal, and sagittal planes.

**Fig 13:**
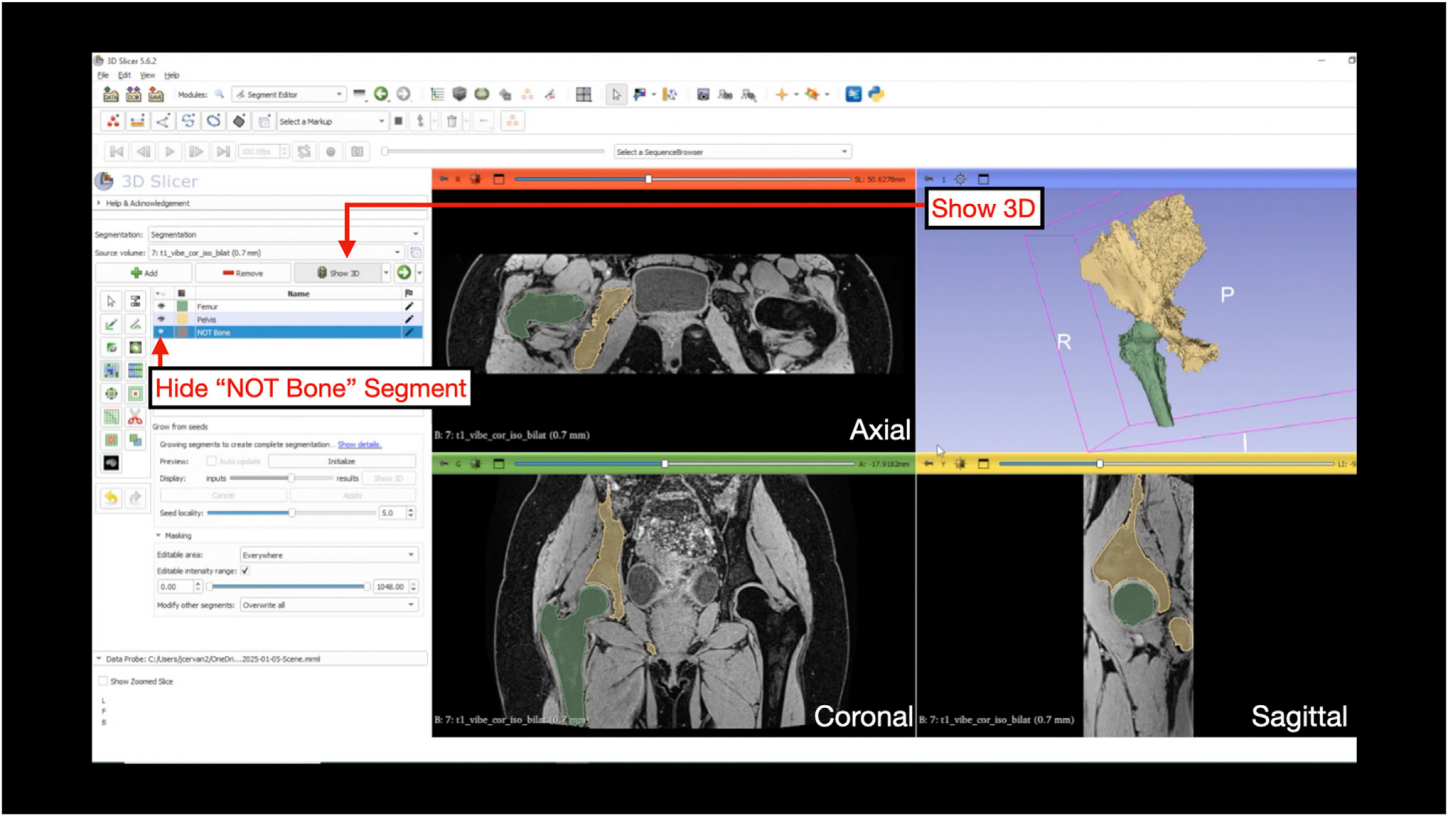
Show 3D. The user can de-select viewing the NOT Bone segment and select Show 3D. This will allow the user to view the segments of interest. The 4-panel view is shown highlighting the course 3-dimensional model generated after applying the grow from seeds segmentation as well as the associated segmentation in the axial, coronal, and sagittal planes.

**Fig 14:**
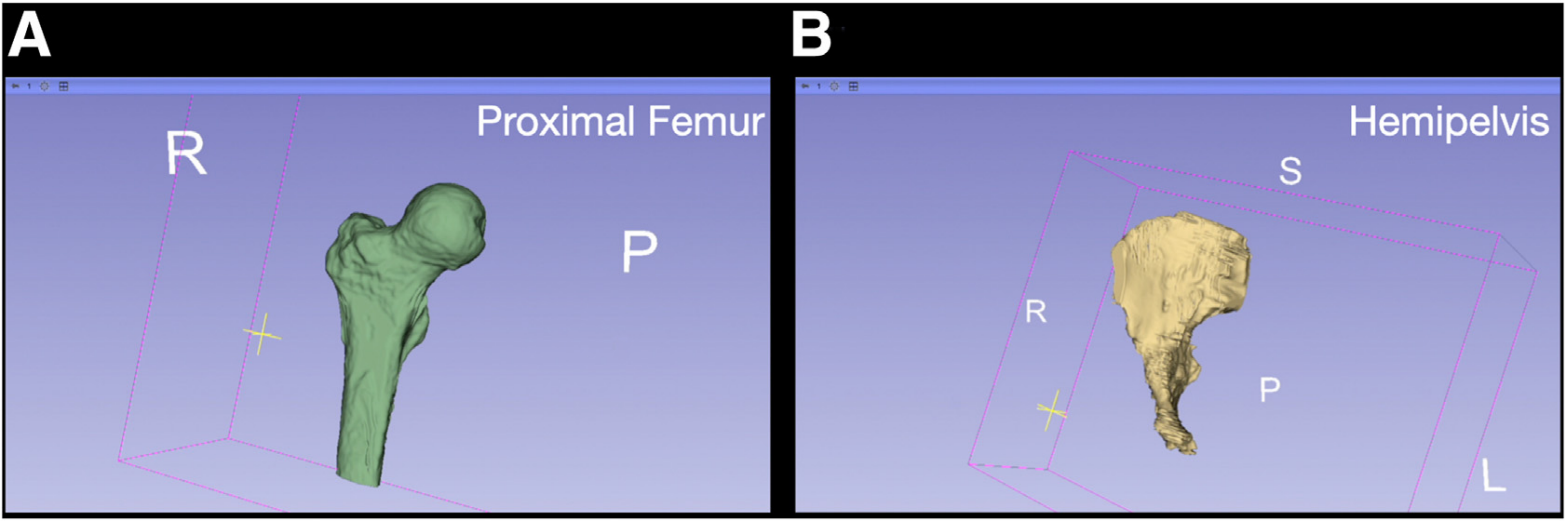
Course 3D-Models of the proximal femur (A) and hemipelvis (B). After refinement of the course 3D-model, the model is viewed prior to cleaning and smoothing steps. The (A) proximal femur is viewed in green from anteromedial view. The (B) hemipelvis is viewed in yellow from anteromedial view.

Under the “Segment Editor” module, the "Grow from Seeds" function provides an automated region of growth based on the manual inputs created. Select “Initialize” and then “Show 3D.” This step may take several minutes, depending on available computational power (Fig 11). The seed locality can be adjusted depending on the results after clicking “Show 3D” by sliding the display slider to visualize the predicted model. “Auto-update” can be selected, which will update the model as the segmentation is edited (note, if using a workstation with less computational power, enabling “Auto-update” may lead to crashing and software lag) (Figs 12 and 13). At this stage, the paint and eraser tool can be used to refine mask boundaries precision. Once the 3D-models is acceptable, click “apply” to finalize the model (Fig 14).

### Smoothing

**Fig 15:**
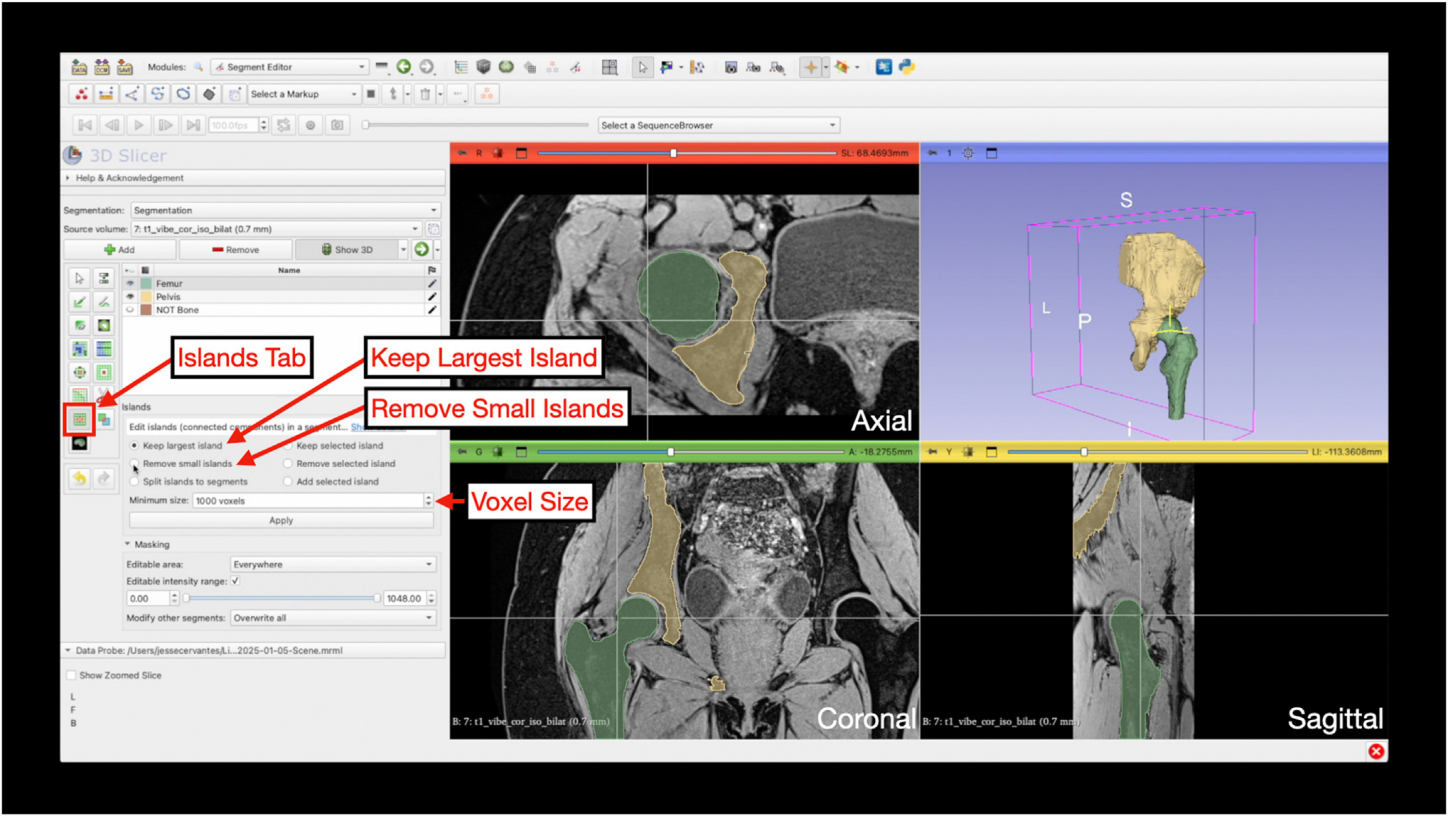
Keeping the largest island and removing small islands. Under the “Segment Editor” module, the Islands tab can be selected. This step allows for artifacts to be eliminated prior to smoothing. For both keeping the largest island and removing small islands, the minimum size can be set to 1,000 voxels. The 4-panel view is shown highlighting the cleaned 3-dimensional model generated after keeping the largest island and removing small islands as well as the associated segmentation in the axial, coronal, and sagittal planes.

**Fig 16:**
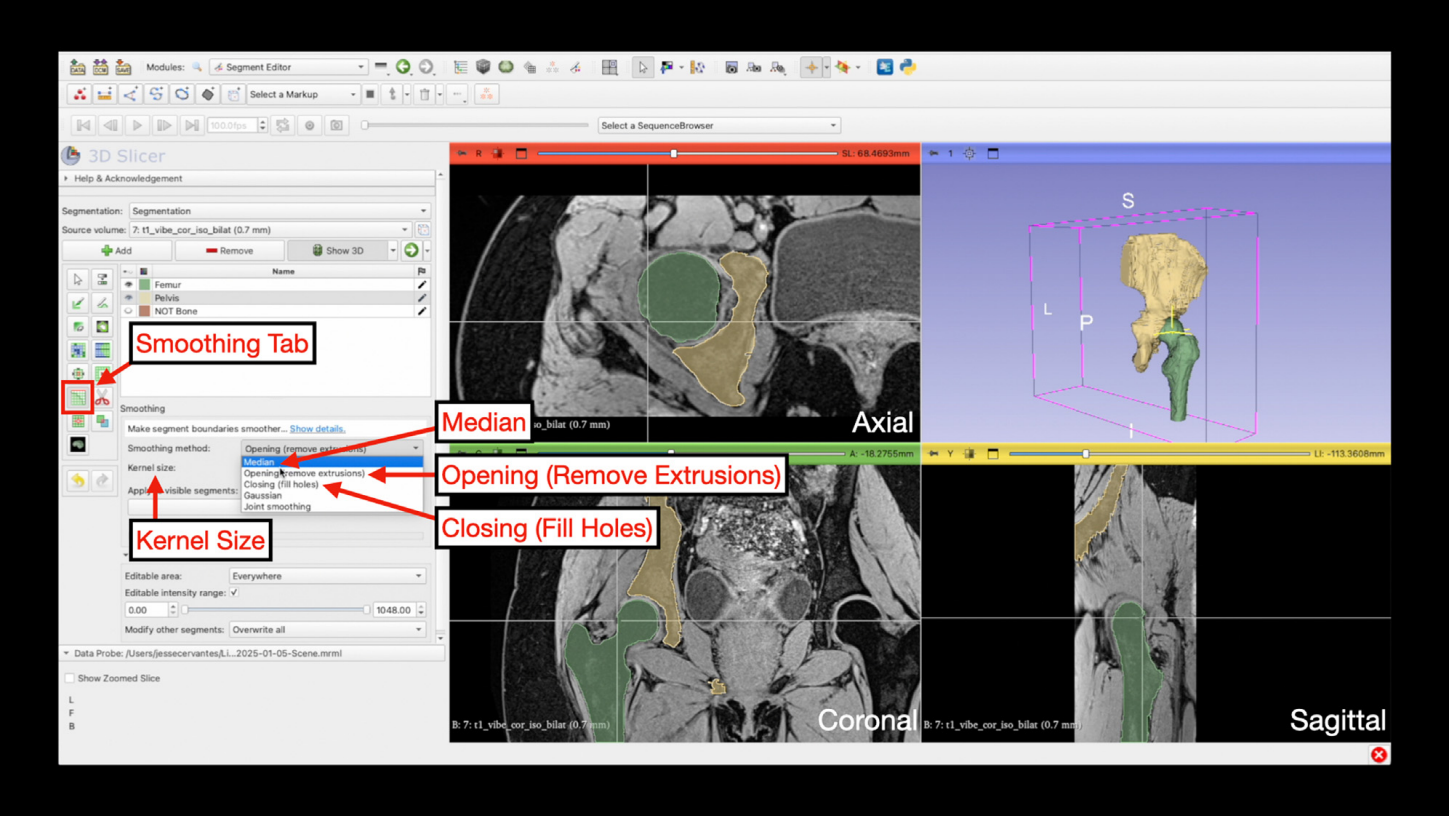
Smoothing. Under the “Segment Editor” module, the Smoothing tab can be selected. This step will smooth the 3-dimensional model to accurately. There are multiple smoothing options, including median, opening, and closing smoothing. The kernel size can be altered for each smoothing step. In sequence, the femur and pelvis can be smoothed using closing at a kernel size of 5 mm, opening at a kernel size of 5 mm, and median at a kernel size of 3 mm. The 4-panel view is shown highlighting the cleaned 3-dimensional model before smoothing as well as the associated segmentation in the axial, coronal, and sagittal planes.

**Fig 17:**
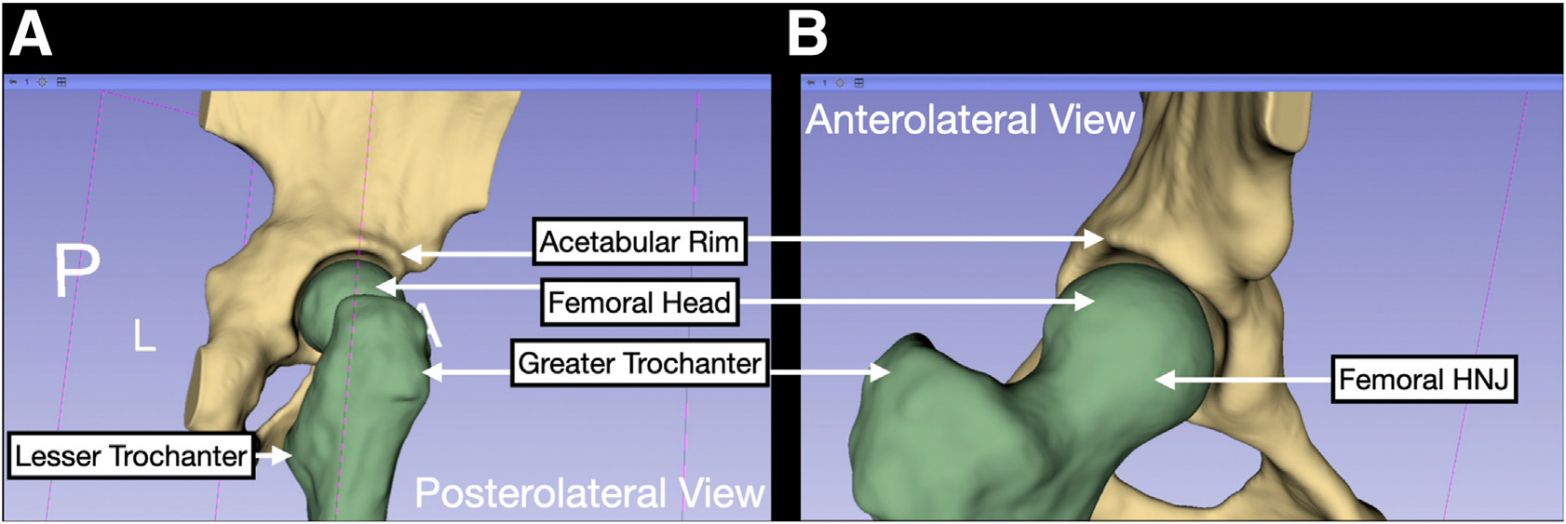
Completed 3-dimensional reconstruction of the proximal femur and hemipelvis. Upon completion of the smoothing steps, the hip joint can be viewed a final time to ensure there are no artifacts. Posterolateral (A) and anterolateral (B) view of the 3-dimensional model are shown illustrating landmarks, including the acetabular rim, femoral head, greater trochanter, lesser trochanter, and femoral head-neck junction (HNJ).

Before smoothing, remove erroneous islands by selecting “Keep Largest Island” and “Remove Small Island” under the “Islands” tab (Fig 15). Under the “Smoothing” tab, there are various options to smooth the 3D-model. For hips, a combination of closing, opening, and median smoothing yields optimal results (Fig 16). Smoothing starts with “Closing (fill holes)” and “Opening (remove extrusions),” both at 5.00-mm kernel size. Smoothing is completed with “Median smoothing” at 3.00-mm kernel size (Fig 17).

### Saving .stl Files

The completed 3D model can be saved as an .stl file under the “Export to files” tab in the “Segmentations” module. Ensure that the NOT Bone mask is hidden such that only the Femur and Pelvis masks are showing by checking “Visible segments only.” Click “Export” to save the .stl file.

### Tips to Overcome Common Issues of Hip Segmentation

When painting over the cortical areas of the proximal femur and pelvis, the curvature of certain anatomical areas (e.g., intertrochanteric line, etc.) may be difficult given the pixel size of the standard paint brush in 3D slicer. To overcome this, changing the oversampling factor to 2.0 allows for a greater resolution when painting (Table 1).

**Table 1:** Pearls and Pitfalls of Hip Joint Segmentation Using 3D Slicer

Pearls	Pitfalls
T1 Vibe sequences from 3-T MRI provide exceptional bone-soft tissue contrast	T1 Vibe sequences from 3-T MRI not routinely acquired clinically
Segmenting multiple sequences using axial, coronal, and sagittal planes increases accuracy of “grow from seeds” function	Segmenting multiple sequences increasing time requirement to complete modeling process
Adjust brush size and increase oversampling factor for complex, curved anatomy	Oversized brush sizes may unintentionally paint over soft tissue
Use “grow from seeds” to accelerate segmentation and time to complete modeling process	Poorly painted segmentations may degrade seed placement after “grow from seeds” function
Take time to learn the 3D Slicer software and consistently save progression of segmentation	Learning curve for users unfamiliar with 3D Slicer software

2D, 2-dimensional; 3D, 3-dimensional; 3T, 3-Tesla; FAIS, femoroacetabular impingement syndrome; MRI, magnetic resonance imaging.

When segmenting the areas listed as not the target femur and pelvis, we use a segmentation listed as “NOT Bone.” Common areas of difficulty include the articular cartilage of the femur/acetabulum and the fovea. By constantly scrolling through slices in all planes with the crosshair feature (which can be accessed in the ribbon, or by holding shift on a point of interest), these areas can be segmented more accurately.

## Discussion

The present technique demonstrates a reproducible methodology to generate high-quality 3D-models of the hip joint from MRI sequences using 3D Slicer. With 3D imaging essential for assessing and planning arthroscopic hip surgery for FAIS and labral tears, the application of reconstructive software with non-ionizing imaging modalities is increasingly important. Software such as 3D Slicer may potentially eliminate various limitations of MRI, such as suboptimal bone visualization in comparison to computed tomography scans.^7^^,^^11^

This technique provides various advantages, namely the ability to generate 3D-models from MRI sequences (Table 2). While we provide the presented technique for the hip, this process can be applied to any joint.^12^ These models allow for a more comprehensive assessment of FAIS morphology than 2D radiographs. In addition they can be applied to assess other pathologies, such as osteoarthritis, dysplasia, or congenital pathologies, such as slipped capital femoral epiphyses.^13^ Although this technique focuses on segmenting the femoral and acetabular bone, additional segments can include the articular cartilage and fibrocartilaginous labrum, which can also be visualized using T1 Vibe sequences from a 3-T MRI. This technique can also be reproduced using 1.5-T MRIs as well, particularly with the use of postprocessing flash-Dixon sequences. However, resolution of articular cartilage and labrum can be limited on a 1.5-T MRI scan compared to on a 3-T MRI scan, and high-resolution sequences are typically obtained for research, rather than clinical, purposes.

**Table 2:** Advantages and Disadvantages of MRI-Based Modeling Using 3D Slicer

Advantages	Disadvantages
Comprehensive assessment of FAIS morphology in comparison to 2D radiographs	Dependent on MRI quality and field strength
Use of MRI avoids ionizing radiation of CT, but technique allows for 3D reconstructions from MRI	Lower spatial resolution for bone compared with CT
Superior visualization of soft tissues, such as labrum and articular cartilage, for segmentation	Longer segmentation times if acquiring soft tissue structures
Clinical and research applications of 3D modeling	Manual segmentation introduces user variability
Open source platform increases availability and accessibility to users	Steep learning curve for new users
Reproducible technique for 1.5-T MRI sequences	
Reproducible technique for other anatomic joints and hip pathologies	

1.5T, 1.5-Tesla; 2D, 2-dimensional; 3D, 3-dimensional; 3T, 3-Tesla; CT, computed tomography; FAIS, femoroacetabular impingement syndrome; MRI, magnetic resonance imaging.

In addition to clinical applications, 3D models can also be used in research settings. Previous literature demonstrates variations in outcomes based on patient-specific factors, such as comorbid conditions.^14^^,^^15^ The generation of a 3D model library can be applied to investigate FAIS morphology in various populations, assess longitudinal progression of disease, and develop automated segmentation systems.^16^ Because software like 3D Slicer is open source, it is widely available and accessible to hip preservation research groups.

## Disclosures

The authors declare the following financial interests/personal relationships which may be considered as potential competing interests: S.J.N. reports consulting or advisory with Stryker; board membership with Arthroscopy Association of North America and American Orthopaedic Society for Sports Medicine; and patents with royalties paid to Stryker, Ossur, and Springer Publishing. All other authors (J.E.C., S.P.M., S.J.N., C.Y.) declare that they have no known competing financial interests or personal relationships that could have appeared to influence the work reported in this paper.

## Supplementary Data

Video 1 The segmentation, cleaning, and smoothing process to create a 3-dimensional (3D) hip joint model reconstruction from a 3-T magnetic resonance imaging series using 3D Slicer, which is a free, open access software. The video highlights necessary steps to import DICOM files, de-identify DICOM files, create segmentations, apply thresholding ranges, paint segmentations, grow from seeds, clean the course 3D-model, smooth the final 3D-model, and export the final .stl file. (DICOM, Digital Imaging and Communications in Medicine.)
